# Online monitoring of protein refolding in inclusion body processing using intrinsic fluorescence

**DOI:** 10.1007/s00216-024-05249-1

**Published:** 2024-04-04

**Authors:** Chika Linda Igwe, Don Fabian Müller, Florian Gisperg, Jan Niklas Pauk, Matthias Kierein, Mohamed Elshazly, Robert Klausser, Julian Kopp, Oliver Spadiut, Eva Přáda Brichtová

**Affiliations:** 1 Competence Center CHASE GmbH, Hafenstraße 47-51, Linz, 4020 Austria; 2 Research Area Biochemical Engineering, Institute of Chemical, Environmental and Bioscience Engineering, Technische Universität Wien, Gumpendorferstraße 1A, Vienna, 1060 Austria; 3 Christian Doppler Laboratory for Inclusion Body Processing 4.0, Institute of Chemical, Environmental and Bioscience Engineering, Technische Universität Wien, Gumpendorferstraße 1A, Vienna, 1060 Austria

**Keywords:** Inclusion body, Protein refolding, Tryptophan and tyrosine fluorescence, Process analytical technology (PAT), Mechanistic model

## Abstract

**Abstract:**

Inclusion bodies (IBs) are protein aggregates formed as a result of overexpression of recombinant protein in *E. coli*. The formation of IBs is a valuable strategy of recombinant protein production despite the need for additional processing steps, i.e., isolation, solubilization and refolding. Industrial process development of protein refolding is a labor-intensive task based largely on empirical approaches rather than knowledge-driven strategies. A prerequisite for knowledge-driven process development is a reliable monitoring strategy. This work explores the potential of intrinsic tryptophan and tyrosine fluorescence for real-time and in situ monitoring of protein refolding. In contrast to commonly established process analytical technology (PAT), this technique showed high sensitivity with reproducible measurements for protein concentrations down to 0.01 g L^-1^. The change of protein conformation during refolding is reflected as a shift in the position of the maxima of the tryptophan and tyrosine fluorescence spectra as well as change in the signal intensity. The shift in the peak position, expressed as average emission wavelength of a spectrum, was correlated to the amount of folding intermediates whereas the intensity integral correlates to the extent of aggregation. These correlations were implemented as an observation function into a mechanistic model. The versatility and transferability of the technique were demonstrated on the refolding of three different proteins with varying structural complexity. The technique was also successfully applied to detect the effect of additives and process mode on the refolding process efficiency. Thus, the methodology presented poses a generic and reliable PAT tool enabling real-time process monitoring of protein refolding.

**Graphical abstract:**

Real-time intrinsic fluorescence monitoring in protein refolding
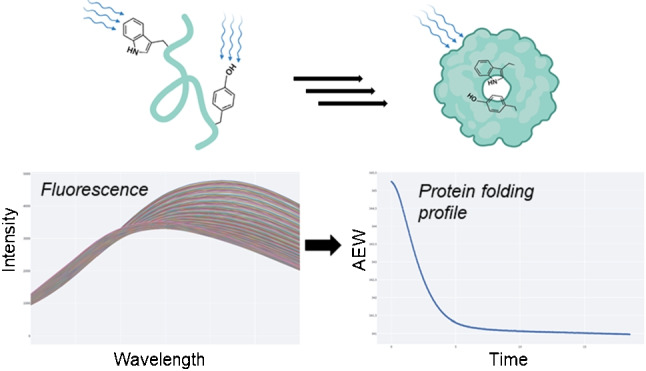

**Supplementary Information:**

The online version contains supplementary material available at 10.1007/s00216-024-05249-1.

## Introduction

Inclusion bodies (IBs) are insoluble protein aggregates formed as a result of protein overexpression in a bacterial host organism. The production of recombinant proteins in form of IBs is a beneficial strategy due to the high yields and purity of the protein of interest. In addition, IBs have a high proteolytic and thermal stability which simplifies their storage between the manufacturing steps [1–4]. The way from production of IBs to correctly folded protein includes several steps: isolation of IBs from cells, their solubilization to unfold the inactive protein structures, a refolding step to obtain the native conformation of the protein before the concentrating and final purification steps [5, 6].

Challenges in protein refolding arise from the highly complex dynamics of the system resulting from fast interconversions coupled to the possibility of multiple transient folding states [7, 8]. A major problem occurring in refolding is protein aggregation which leads to the formation of misfolded aggregates rather than bioactive correctly folded protein [9, 10]. To avoid undesired protein aggregation, refolding is usually carried out at low protein concentrations [11]. Consequently, the process is typically conducted in batch dilution mode [11, 12] using an optimized buffer composition often including chemical additives to further suppress the aggregation reaction [6, 13, 14]. Process knowledge in combination with systematic strategies is essential to reduce the effort of state-of-the-art empirical process development and optimization [15, 16]. Common strategies include buffer optimization via Quality-by-Design principles [17, 18] or optimization of the processing mode [19] by incorporating model-based approaches [20].

In an industrial environment, real-time monitoring and control strategies, collectively referred to as process analytical technology (PAT), of protein refolding are highly desirable to avoid deviations in the process performance and subsequently the product quality [21]. However, in protein refolding suitable real-time monitoring and control strategies are scarce. As protein concentrations in batch approaches are mostly far below 1 g L^-1^, commonly established online and inline sensors are not sensitive enough to track the changes in the process or even detect those concentrations [12]. Changes in folding states are commonly analyzed using either chromatographic methods [22, 23] or via measurements of the biological activity [18]. However, these techniques cannot be implemented in the online mode due to their instrumentation requirements. Additionally, they often require tedious sample preparation. Spectroscopic methods have shown to be promising for monitoring of structural changes in the protein stability and aggregation studies [23–25], however, their potential to monitor protein refolding has not been fully unlocked [6, 26].

Intrinsic tryptophan (Trp) and tyrosine (Tyr) fluorescence is a well-established method for the observation of different folding states of proteins [26, 27], especially in the field of protein stability and ligand binding [25, 28]. Trp and Tyr fluorescence is sensitive to the polarity of the local environment of these residues [29]. Along the various folding states of a protein, the exposure level of the hydrophobic Trp and Tyr side chains decreases from denatured to native state. Subsequently, the polarity of the local environment also changes leading to shifts in the maximum of the emission spectrum and changes in the signal intensity [28, 29]. Although intrinsic Trp and Tyr fluorescence is widely used to study the protein stability and aggregation [30–32], its application in IB processing is still scarce. Sharma et al. [33] used measurements of the intrinsic fluorescence in the segmented-based optimization of protein refolding conditions for a fragment antigen-binding region of an antibody. By offline measurement of samples from various stages of refolding process, they attempted to correlate the observed shifts in the maximum wavelength of Trp and Tyr fluorescence spectrum to the progress of refolding process [33]. However, in their work, Trp and Tyr fluorescence monitoring has been used only in the offline mode and has not been applied continuously in the entire process duration; therefore, it was not possible to extract information about process kinetics.

As protein refolding is of a great industrial interest, model-based approaches to the process are highly desirable to reduce the optimization time and costs. However, for protein refolding processes there are only few model-based applications since the availability of online measurements is strongly limited [12]. The most widely used mechanistic model for the description of refolding dynamics reduces the complexity of refolding reactions to four model states while encountering the difference in reaction rates of aggregation and refolding [9, 10]. It is known that the reaction rates of refolding ($k_N$) and aggregation ($k_A$) are changing depending on the concentration of the denaturing agent [34]. In addition, the aggregation reaction is often assumed to be of second order [10]. Still, it is known that progression of the dynamics is mainly depending on the refolding intermediates which are known to be the reactive species [35]. Time-resolved online monitoring would be a valuable technique in order to expand the knowledge of refolding kinetics further and build model-based applications.

In this work, we present an implementation of continuous in situ monitoring of protein refolding processes based on intrinsic Trp and Tyr fluorescence. The versatility of the implemented method is demonstrated on the refolding of three enzymes — lactate dehydrogenase (LDH), galactose oxidase (GalOx) and horseradish peroxidase (HRP). During the refolding processes two parameters of the fluorescence emission spectra obtained after excitation at 280 nm were monitored: average emission wavelength (AEW), i.e., the center of mass of a fluorescence peak, and the integral of fluorescence intensity over the wavelength range measured. We showed that the AEW profile over the refolding process is decreasing exponentially for most of the monitored processes which can be related to the decrease in the amount of folding intermediates during the process. Key performance indicators (KPIs) of refolding were obtained from the exponential curve fit for each process to make a more quantitative comparison of the processes. LDH refolding was investigated with respect to different additives and processing modes. Intrinsic Trp and Tyr fluorescence was also shown to be applicable to refolding processes requiring the addition of a cofactor during the process, as shown for GalOx and HRP refolding. Moreover, the data obtained from the continuous monitoring of fluorescence were employed as an input for a mechanistic refolding model which opens up the way for implementation of model-based approaches utilizing fluorescence monitoring.

## Materials and methods

### Proteins

L-Lactate dehydrogenase 1 (LDH), galactose oxidase (GalOx) and horseradish peroxidase (HRP) were produced as IBs in *E. coli* cultivations. A monomeric LDH originating from *Lactobacillus plantarum* is with a size of 34.4 kDa and no disulfide bridges was used in this work. The molecular weight of GalOx from *Fusarium graminearum* is approximately 68.5 kDa and the enzyme incorporates two disulfide bridges. A thioether-cross-link is formed upon addition of copper as a cofactor [36]. HRP is an oxidoreductase containing a heme cofactor. HRP C1A isoenzyme with a size of 34.5 kDa and four disulfide bridges was used in this work.

### Production of IBs

Production of LDH IBs was performed as described in [37]. In brief, *E. coli* BL21(DE3) cells were cultivated in DeLisa minimal medium [38]. Main cultures were carried out in fed-batch mode in a 3.3-L Labfors bioreactor (Infors AG, Bottmingen, Switzerland) with controlled feeding at a specific glucose uptake rate (q_s_) of 0.2 g g^-1^ h^-1^. The culture was induced with 1 mM isopropyl-D-thiogalactopyranoside (IPTG). Induction was carried out for 6 h at 37°C and a q_s_ of 0.25 g g^-1^ h^-1^. IBs were separated from the harvested biomass by high-pressure homogenization and washing steps as described elsewhere [37].

IBs of GalOx were produced in *E. coli* BL21 (DE3) cultures using a pET-29b(+) vector and a T7-expression system. Pre-cultures were grown in DeLisa pre-culture medium [38] supplemented with 50 µg mL^-1^ kanamycin and 8.8 g L^-1^ glucose. Baffled shake-flasks with a filling volume of 500 mL were inoculated with 0.5 mL cryo-preserved culture and cultivated (37°C, 16 h, 250 rpm) in an Infors HR Multitronshaker (Infors AG, Bottmingen, Switzerland). Cultivation in a 15-L Biostat^®^ Cplus stainless steel reactor (Sartorius, Göttingen, Germany) was conducted in three phases: first, a batch phase was initiated by the inoculation of 4500 mL of DeLisa batch medium [38] supplemented with 50 µg mL^-1^ kanamycin and 15.5 g L^-1^ glucose with 500 mL of the pre-culture. Then, a fed-batch phase with a specific glucose uptake rate (q_s_) of 0.25 g g^-1^ h^-1^ and a feed concentration of 440 g L^-1^ was followed by an induced fed-batch phase with a q_s_ of 0.20 g g^-1^ h^-1^ that was initiated by the addition of 1 mM IPTG. The temperature was controlled via the heating jacket of the vessel, being 35°C during the first two phases, and 30°C after induction. Adjustment of stirrer speed (500–900 rpm), oxygen mole fraction in the gas flow (20.95–24.11% (v/v)), and pressure (0.5–1.0 bar) were used to maintain the dissolved oxygen tension (DOT) above 40%. A constant pH 6.9 was controlled by the addition of 2 M phosphoric acid and 12.5% ammonium hydroxide (v/v). The biomass was harvested 6 h after induction by centrifugation of the cell suspension (16,000x g, 25 min, 4 °C). The resulting cell pellet was stored at -20 °C until further processing. The cell rupture and IB isolation were conducted as described elsewhere [37].

The production of HRP IBs was previously described in Humer et al [18]. Briefly, the hrp gene coding for HRP variant C1A was codon-optimized for *E. coli* and obtained from GenScript USA Inc. (Piscataway, NJ, USA). The plasmid pET21d+ was used for HRP IB production in the cytoplasm. A stop codon was introduced to produce HRP without any tags. HRP was produced in *E. coli* BL21(DE3) in a 10-L Biostat Cplus stainless steel bioreactor (Sartorius, Germany). The pre-culture was grown in 0.5 L DeLisa medium [38] at 37 °C, 230 rpm in a 2.5-L Ultra Yield™ Flask (UYF; Thomson Instrument company, Encinitas, CA, USA) over night. Subsequently, the pre-culture was added to 4.5 L DeLisa medium [38] in the bioreactor vessel and batch fermentation at 35 °C was run for 6 h. The pH was maintained at 7.2 and the DOT was kept above 20%. During the 16 h fed-batch phase q_s_ was 0.333 g g^-1^ h^-1^, which was set to 0.25 g g^-1^ h^-1^ after induction with 0.5 mM IPTG. After an induction phase of 8 h, the biomass was harvested by centrifugation. IBs were separated from the harvested biomass by high-pressure homogenization and washing steps [18].

### Processing of IBs — solubilization

The solubilization of IBs of LDH and GalOx was performed at a concentration of 100 g IB wet weight L^-1^ at 25°C under slight agitation for 2 h. The resulting suspension was then centrifuged (20,000x g, 4°C) before the supernatant was stored at 4°C until further processing. LDH IBs were solubilized in a buffer containing 150 mM NaH_2_PO_4_, pH 6.0, 4 M GuHCl. GalOx IBs were first mixed with solubilization buffer — 150 mM NaH_2_PO_4_, pH 7.0, 6 M GuHCl, before dithiothreitol (DTT) was added at a concentration of 25 mM to initiate the disruption of disulfide bonds. DTT stocks at a concentration of 1 M were prepared freshly before the adequate volume was added to the solubilization buffer. Concentrations of the solubilized protein were determined before refolding was initiated in a batch dilution approach. IBs of HRP were solubilized at a concentration of 100 g IB wet weight L^-1^ in a buffer containing 6 M urea, 7.11 mM DTT, 50 mM glycine at pH 10 for circa 0.5 h at 4–10°C under slight agitation [18]. The resulting suspension was then centrifuged (20,000x g, 4°C) for 20 min.

### Processing of IBs — refolding

Refolding was conducted at 5°C for LDH and GalOx and 7.5°C for HRP with a constant stirring between 500 and 800 rpm. To initiate the refolding process, the solubilized protein was rapidly added into pre-cooled refolding buffer directly inside the fluorescence cuvette (“Online intrinsic fluorescence monitoring”). The samples were incubated at constant temperature and stirring speed for 2.5 h for LDH and GalOx or 22 h for HRP.

#### Refolding of LDH

The solubilized LDH was refolded in a buffer containing 150 mM NaH_2_PO_4_ at pH 6.0 unless stated otherwise.

Different additives (L-arginine, acetone and glycerol) and the addition of excess GuHCl were tested for their effects on aggregate formation. The chemicals were added to the LDH refolding buffer and refolding was carried out in batch processing mode. The detailed experimental design can be found in Table 1.

**Table 1:** Experimental design for buffer screening of additives for LDH refolding

	Additive	Concentration	DF	c_GuHCl_*
Process	-		-	[M]
a	L-arginine	1 M	40	0.10
b	None	–	40	0.10
c	Glycerol	10% (v/v)	40	0.10
d	GuHCl	0.08 M	50	0.16
e	Acetone	1.4 M	50	0.08
f	GuHCl	0.6 M	50	0.68

^∗^Total concentration incl. carry over. *DF*, dilution factor

To investigate the suitability of different processing modes for LDH refolding, 8 experiments were conducted using the standard refolding buffer. The experiments were set up following a full-factorial design-of-experiment (DoE) approach, where the final dilution factor (10, 30, 50) and the number of pulses (1, 3, 5) were altered. Here, a pulse number of 1 refers to batch dilution and higher numbers to pulsed batch processing. Five different conditions were tested, with the center point being conducted as biological replicates ($n = 4$). The complete experimental setup is described in Table 2.

**Table 2:** Experimental design for pulsed LDH refolding

Process	Replicates	DF	Pulses
a	4	30	5
b	1	50	1
c	1	50	3
d	1	10	1
e	1	10	3

*DF*, dilution factor

#### Refolding of GalOx

Refolding of solubilized GalOx was carried out in a buffer containing 100 mM NaH_2_PO_4_, 5 mM cystamine, 1 M L-arginine at pH 7.4 unless stated otherwise. Refolding was carried out in batch mode at varying dilution factors. Cu^2+^ was added as a cofactor at a concentration of 1 mM. The time of addition was varied according to the experimental setup described in Table 3. The design was based on a full-factorial DoE approach with the center points being carried out as biological replicates ($n=3$).

**Table 3:** Experimental design for GalOx refolding

Process	Replicates	DF	t_add_
	-	-	[min]
a	3	30	75
b	1	10	30
c	1	50	120
d	1	10	120
e	1	50	30

*t*_add_, process time of copper addition; *DF*, dilution factor

#### Refolding of HRP

Refolding of solubilized HRP was performed in a buffer containing 2 M urea, 2 mM CaCl_2_, 7% (v/v) glycerol, 1.27 mM GSSG — oxidized form of glutathione, at pH 10 [18]. Each measurement was performed in duplicate. The refolding process was initiated by dilution of the solubilized protein in the refolding buffer to the final HRP concentration of 0.5 g L^-1^. The refolding process was monitored over 20 h at 7.5°C with a constant stirring of 800 rpm. After 20 h, hemin cofactor (1 mM stock in 100 mM KOH solution) was added to the refolding samples to reach the hemin concentration of 5 $\mu$M or 20 $\mu$M in the samples. After the hemin addition, the samples were monitored for additional 2 h at 7.5°C with a constant stirring of 800 rpm.

### Online intrinsic fluorescence monitoring

Intrinsic Trp and Tyr fluorescence was measured using an FP-8550 Spectrofluorometer (Jasco, Tokyo, Japan) with a multi-cuvette holder (Jasco, Tokyo, Japan) enabling thermostating and stirring of the cuvettes. Refolding was carried out in 3-mL quartz fluorescence cuvettes (Starna GmbH, Germany) with magnetic stirrers at a volume of either 1.5 mL or 3.0 mL. The temperature of the cuvette holder was set to 5°C (LDH, GalOx) or 7.5°C (HRP) and the stirring speed was set between 500 and 800 rpm. The sample was excited at 280 nm and the emission spectrum was recorded between 310 and 370 nm with a step size of 0.5 nm. Excitation and emission slits of 1 nm and 10 nm, respectively, were used. The scanning speed was set to 200 nm min^-1^, sensitivity to medium, and the response time to 0.5 s. Data processing was conducted using python 3.7. Pre-processing was carried out by calculating the integral of the intensity *f* from $\lambda _0$ to $\lambda _1$ using Eq. 1.

1 $$\begin{aligned} F(t) = \int _{\lambda _0}^{\lambda _1} f(t) d\lambda \end{aligned}$$

The average emission wavelength (AEW) of the emission spectra was calculated by Eq. 2, where F_i_ is the fluorescence emission intensity at wavelength $\lambda _i$.

2 $$\begin{aligned} AEW = \frac{\sum (\lambda _i \cdot F_i)}{\sum F_i} \end{aligned}$$

For time course measurements, spectra were collected in approximately 1-min intervals between 310 and 370 nm and AEW and fluorescence intensity integrals *F* were calculated for each spectrum. An exponential decay function as described in Eq. 3, was fit to the development of AEW over time, where *y(t)* corresponds to the AEW curve fit over process time (*t*). $y_0$ describes the y-intercept, *k* the exponential decay coefficient in min^-1^ and *d* the final AEW in nm at the equilibrium. Its derivative with respect to time (Eq. 4) was used to assess the reactivity of the process with $\dot{y}(t)$ describing the change of AEW in nm min^-1^. A refolding reaction was considered to be finished when the derivative was below 5% of the maximum rate of change.

3 $$\begin{aligned} y(t) = (y_0-d)\cdot e^{-k\cdot t}+d \end{aligned}$$

4 $$\begin{aligned} \dot{y}(t) = -(y_0-d)\cdot k \cdot e^{-k\cdot t} \end{aligned}$$

The sensitivity of the exponential decay constant *k* was assessed by calculation of the signal-to-noise ratio (SNR) as shown in Eq. 5, with *k* in min^-1^, where $\sigma _k$ is the standard deviation of *k*. *k* was considered to be inconclusive when falling below the threshold of 10$\sigma$ [39].

5 $$\begin{aligned} \text {SNR}_k = \frac{k}{\sigma _k^2} \end{aligned}$$

From Eq. 2$\Delta$AEW in nm is calculated as a function of process time (*t*) in min (Eq. 6).

6 $$\begin{aligned} \Delta AEW(t) = AEW(t)-AEW(t=0) \end{aligned}$$

### Offline analytical tools

#### Quantification of protein concentration

Concentrations of the total protein in the soluble fraction were quantified using reverse-phase high-performance liquid chromatography (RP-HPLC) as described by [40] with a Polyphenyl BioResolve-RP-mAb 2.7 µm 3.0 x 100 mm column (Waters Corporation, Milford, USA) and an UltiMate 3000 HPLC system (Thermo Fisher Scientific, Waltham, MA, USA). Bovine serum albumin was used as a reference standard in a concentration range of 0.05–2.0 g L^-1^. The concentration of insoluble protein was calculated based on the theoretically added total protein subtracted by the amount of soluble protein fraction.

#### Enzymatic activity assay

Enzymatic activities were all measured using photometric assays conducted in a TECAN Spark^®^ microplate reader (Tecan Trading AG, Männedorf, Switzerland). The temperature was set to 30°C and absorbance was recorded for 2 or 3 min.

To measure the enzymatic activity of LDH, the reaction buffer (100 mM NaH_2_PO_4_, 0.425 mM nicotinamide adenine dinucleotide (NADH), 0.45 mM pyruvate) was mixed with sample at a ratio of 30% (v/v). Absorbance was recorded at 340 nm using the extinction coefficient of NADH, which is 6.22 mM^-1^ cm^-1^ [41]. Here, 1 Unit was defined as the necessary enzyme for the conversion of 1 µmol of NADH per minute. The volumetric enzymatic activity (*vAc*) was calculated as follows:

7 $$\begin{aligned} vAc = \frac{V_t \cdot \frac{\Delta A}{\Delta t}}{V_s \cdot l \cdot \epsilon } \end{aligned}$$

Calculation of the volumetric enzymatic activity (v*Ac*) in U mL^-1^ was based on the change of absorbance over time ($\Delta A / \Delta t$). $V_t$ stands for the total volume of the reaction mixture, $V_s$ is the volume of the enzyme solution, *l* is length of the optical path (*l* = 0.62 cm) and $\epsilon$ is the extinction coefficient. The specific activity was calculated by dividing the volumetric activity by the target protein concentration determined via RP-HPLC.

The enzymatic activity of GalOx was determined via a two-stage colorimetric assay, where the reaction of galactose to H_2_O_2_ catalyzed by GalOx is coupled to a chromogenic 2,2^′^-azinobis(3-ethylbenzothiazolinesulfonic acid) (ABTS) assay. In brief, an assay solution consisting of 4% HRP stock solution (0.6 g L^-1^ HRP in 50 mM Tris-HCl, 1 M (NH_4_)_2_SO_4_, pH 7.5), 10% ABTS stock solution (6.125 g L^-1^ ABTS in 0.1 M NaH_2_PO_4_, pH 7.5) and 40% D-galactose (1 M) was prepared in NaH_2_PO_4_ at pH 7.5. Then, the prediluted sample was mixed with the assay solution at a ratio of 30% (v/v). Absorbance was measured at 420 nm using an $\epsilon$ of ABTS of 36 mM^-1^ cm^-1^ [42]. The enzymatic activity was calculated according to Eq. 7. Here, 1 Unit was defined as the necessary enzyme for the oxidation of 2 µmol of ABTS per minute.

HRP activity assay also employs ABTS as a peroxidase substrate. The reaction mixture for the assay of a total volume of 200 µL contained: 175 µL of 8 mM ABTS solution in 50 mM phosphate-citrate buffer pH 5, 20 µL of 10 mM H_2_O_2_ in ultrapure water and 5 µL of the HRP sample after refolding diluted 1:1,000 in 20 mM BisTris/HCl pH 7. Absorbance at 420 nm was measured and the volumetric enzyme activity was calculated using Eq. 7, using the extinction coefficient at 420 nm ($\epsilon _{420}$ = 36 mM^-1^ cm^-1^). The volumetric activity was tested for the samples at the end of monitored refolding process, i.e., after 22 h of refolding. The average volumetric activity and its standard deviation for each process were calculated from 9 independent activity measurements.

#### Circular dichroism

Circular dichroism (CD) spectra were measured in 0.1 cm pathlength SUPRASIL^®^ quartz cuvettes (HellmaAnalytics, Müllheim, Germany) using a J-815 CD Spectrometer (Jasco, Tokyo, Japan). The temperature during the measurement was set to 5°C. The resulting far-UV CD spectrum was obtained as an average of three scans between 200 and 250 nm and the spectrum of the pure buffer was subtracted. Samples were diluted to a protein concentration of 0.5 g L^-1^. The mean residue ellipticity (MRE) in deg cm^-2^ dmol^-1^ was calculated as described in Eq. 8, where $\theta _{obs}$ is the CD in mdeg, *M* is the molecular weight of the protein in g dmol^-1^, *l* is the pathlength in cm, *n* is the number of amino acid residues, and *c* is the protein concentration determined via RP-HPLC in g L^-1^.

8 $$\begin{aligned} MRE = \frac{\theta _{obs} \cdot M}{n \cdot l \cdot c} \end{aligned}$$

### Process model

The refolding process model used was adapted from Kiefhaber et al. [9]. It is composed of three ordinary differential equations (ODEs) describing the state dynamics of intermediates (*I*) (Eq. 9), native protein (*N*) (Eq. 10) and aggregated protein (*A*) (Eq. 11) in a batch type process with a constant volume.

9 $$\begin{aligned} \frac{dI}{dt} = -k_N \cdot I - k_A \cdot I^n \end{aligned}$$

10 $$\begin{aligned} \frac{dN}{dt} = k_N \cdot I \end{aligned}$$

11 $$\begin{aligned} \frac{dA}{dt} = k_A \cdot I^n \end{aligned}$$

The refolding rate of the native protein $k_N$ and the aggregation rate $k_A$ are described by the following algebraic kinetic equations

12 $$\begin{aligned} k_N = a_N\cdot (1+D)^{b_N} \end{aligned}$$

13 $$\begin{aligned} k_A = a_A\cdot (1+D)^{b_A} \end{aligned}$$

with the kinetic model parameters $a_N$, $b_N$, $a_A$ and $b_A$, that can be experimentally identified and *D* representing the concentration of the denaturing agent. For the considered LDH refolding process the parameters have been identified elsewhere [43] and were specified with $a_N={1.33\,\mathrm{\text {h}^{-1}}}\pm {1.58\,\mathrm{\text {h}^{-1}}}$, $a_A={12.05\,\mathrm{\text {h}^{-1}}}\pm {11.36\,\mathrm{\text {h}^{-1}}}$, $b_N={-8.68\,\mathrm{\text {h}^{-1}}}\pm {0.71\,\mathrm{\text {h}^{-1}}}$ and $b_A={-16.78\,\mathrm{\text {h}^{-1}}}\pm {2.57\,\mathrm{\text {h}^{-1}}}$. For the pulsing experiments, discrete events have been introduced where the concentrations of *I* and *D* were increased according to the calculated concentration after pulse addition.

The output functions Eqs. (14) and (15) were used to describe the relationship between the model states and intrinsic fluorescence measurements, where $P = I + N$ and $\beta _1$ to $\beta _5$ being the parameters obtained by the experimental data fit. *P* was considered as the total dissolved protein concentration without considering insoluble aggregates.

14 $$\begin{aligned} F(t) = \beta _1\cdot \frac{P}{\beta _2 + P} \end{aligned}$$

15 $$\begin{aligned} \Delta AEW(t) = \beta _3\cdot \frac{\frac{(N+A)}{P}}{\beta _4 + \frac{(N+A)}{P}} + \beta _5 \cdot \frac{(N+A)}{P} \end{aligned}$$

### Modeling framework

The programming language Julia was used for the model analysis and simulation. The model was defined symbolically using ModelingToolkit.jl [44] and the ODE system was numerically solved using DifferentialEquations.jl [45] from the Julia SciML ecosystem.

## Results and discussion

### Online monitoring of LDH refolding

**Fig. 1:**
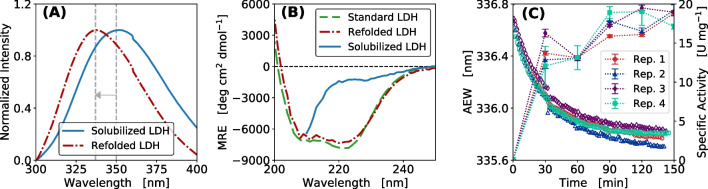
Conformational change of LDH during protein refolding. (**A**) Fluorescence emission spectra of refolded and solubilized LDH after excitation at 280 nm. Fluorescence intensity was normalized between 0 and 1. (**B**) Far-UV CD spectra of refolded LDH, solubilized LDH and commercially available LDH protein standard derived from porcine muscle (CAS No. 9001-60-9). The far-UV CD signal was converted into concentration independent mean residue ellipticity. (**C**) AEW and specific activity of LDH batch refolding, with the dilution factor of 40, as four biological replicates (Rep.1–4). AEW in nm and specific activity in U mg^-1^ were measured over the process time (*k* = 0.032 ± 0.002 min^-1^, $\Delta ~AEW$ = 0.86 ± 0.05 nm). Measurements of the specific activity are shown as technical replicates ($n=3$)

In Fig. 1 conformational changes of LDH refolding were analyzed by comparing CD spectra to measurements of intrinsic Trp and Tyr fluorescence of solubilized and refolded samples. The shift in AEW obtained after 2.5 h of four LDH batch refolding processes was compared to the offline measurements of enzymatic activity to investigate the suitability of intrinsic Trp and Tyr fluorescence for monitoring of protein refolding.

**Table 4:** Comparison of KPIs of LDH batch refolding using different buffer compositions

	Additive	$\Delta$AEW	F_0_	*k*	SNR_k_	Specific Activity	Aggregation	t_end_
Process	-	[nm]	-	[min^-1^]	-	[U mg^-1^]	[g g^-1^]	[min]
a	1 M L-arginine	0.12	249,212	0.12	9	0.03 ± 0.02	0.08	ND
b	–	0.8	532,224	0.0214	39	20.45 ± 0.07	0.15	141
c	10% glycerol	0.58	565,166	0.0188	40	7.80 ± 0.28	0.53	160
d	0.08 M GuHCl	0.84	172,000	0.0351	51	23.91 ± 0.35	ND	86
e	1.4 M acetone	ND	711	0.0012	0.2	ND	0.57	ND
f	0.6 M GuHCl	0.38	237,640	0.0293	21	ND	0.03	103

*ND*, not detectable; *t*_end_, mathematically defined end of refolding, i.e., below 5% of the maximum rate of change of AEW

Both intrinsic fluorescence and far-UV CD spectra of solubilized LDH and the sample after refolding reflect the changes in the protein conformation (Fig. 1A and B). The fluorescence spectra in Fig. 1A show that the maximum emission wavelength of refolded LDH after Trp and Tyr excitation was at 337 nm while the solubilized LDH had its maximum at 350 nm. The conformational change between solubilized (denatured) LDH and LDH after refolding is characterized by a total shift of fluorescence maximum of 13 nm. The far-UV CD spectra (Fig. 1B) show that the refolded LDH had a similar structure as the commercially available standard derived from porcine muscle (CAS No. 9001-60-9). The refolded protein and the protein standard showed local minima at 222 and 208 nm being typical characteristics for proteins with a high content of $\alpha$-helical structure [46]. In contrast, the solubilized protein, which is believed to be completely unfolded, showed a pattern that is typical for random coils [46]. In Fig. 1C online measurements of the AEW over the duration of a batch refolding processes showed fast dynamics during the first 30 min which was reflected as a steep decrease in AEW. Afterwards the change in AEW per minute decreased resulting in an almost constant signal after 120 min, with a final shift of AEW by 0.86 nm. The inverse behavior could be observed when monitoring the specific activity over processing time (Fig. 1C). Here, 80% of the final activity was reached after 30 min. This similar progression of the curves depicts the suitability of using intrinsic fluorescence measurements to monitor the course of protein refolding reactions. Comparing four biological replicates, the results showed minor deviations of ±0.002 min^-1^ and ±0.05 nm within the exponential decay coefficients and the total shifts in AEW, respectively, thereby highlighting the reproducibility of the method.

### Effect of additives on LDH refolding

To show the usability of Trp and Tyr fluorescence monitoring for process development in protein refolding, Table 4 compares the key performance indicators (KPIs) of multiple LDH batch refolding processes with different chemical additives to the change in AEW and its exponential decay. The corresponding plots of intensity integral and AEW over the process time are given in Fig. S2.

**Fig. 2:**
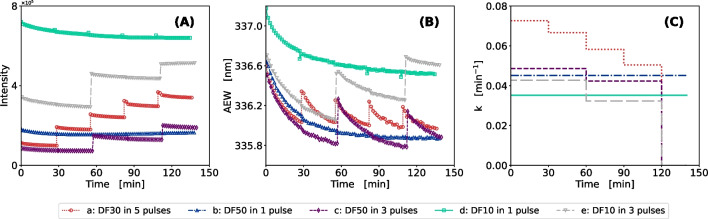
Comparison of batch and pulsed batch mode for LDH refolding. Five different process modes (a–e) with variations in final dilution factor (DF) and number of pulse additions were compared regarding (**A**) fluorescence intensity over process time, (**B**) average emission wavelength in nm over process time, and (**C**) k in min^-1^ over process time. Experiments were conducted in a DoE approach with the center point (process a) as biological replicates ($n=4$)

Various additives have been investigated regarding their suitability to prevent aggregation in LDH refolding. Table 4 shows that the highest specific activity was reached for the process without additives (process b) and for process d with the addition of 0.08 M GuHCl. In both cases the total shift in AEW exceeded 0.8 nm with reaction rates of 0.021 and 0.035 min^-1^ for processes b and d, respectively. L-arginine is one of the most used aggregation inhibitors [13, 14] and its addition to LDH refolding indeed decreased aggregation to a minimum (Table 4, process a). However, indicated by a shift of AEW of only 0.12 nm and a low specific activity suggest also almost no LDH refolding. As arginine is known to stabilize folding intermediates by promoting protein solvation while simultaneously acting as a denaturing agent [6], we assume that a concentration of 1 M of arginine was too high, inhibiting both refolding and aggregation. This is also indicated by the low SNR_k_. A similar result was obtained for process f with the addition of 0.6 M GuHCl. Here, a final shift in AEW of 0.38 nm corresponds to the absence of activity and aggregate formation. Still, as there was a measurable shift in AEW, we suspected that there was a conformational change towards more energetically favorable intermediates. However, the concentration of denaturing agent was too high to enhance the transition towards the bioactive form of LDH. These results represent the importance to optimize the concentration of the denaturing agent in refolding, as it is the key to enhanced product recovery [35]. The addition of acetone (process e) in refolding led to an instantaneous precipitation of the solubilized protein and loss of the fluorescence signal (Table 4). Consequently, the absolute change in AEW and *k* cannot be determined for the process. However, this immediate response of the fluorescence intensity shows the potential to employ changes of fluorescence intensity for observation of insoluble aggregation as insoluble aggregates contribute less to the signal when precipitating out of solution.

Table 4 illustrates trends between changes in the fluorescence signal and the refolded product and aggregate formation. Low shifts in AEW (<0.6 nm) correlate with the low extent of refolding and aggregation, while higher shifts ($\ge$0.6 nm) either indicate higher concentration of native protein, aggregates or both. Consequently, online measurements of the intrinsic fluorescence enable better understanding of the process. In combination with small-scale and parallel experimental setups, as it was demonstrated here, they can be beneficial for buffer screening experiments. The combination with systematic approaches or model-based experimental planning can be another step towards more knowledge-driven strategies in process development.

**Table 5:** Comparison of KPIs of LDH pulsed refolding

Process	DF	GuHCl conc.	$\Delta$AEW*	Specific activity
	-	[M]	[nm]	[U mg^-1^]
a	30	0.13	1.74	10.7 ± 0.20
b	50	0.08	0.76	10.0 ± 0.19
c	50	0.08	1.59	18.4 ± 0.01
d	10	0.4	0.65	3.7 ± 0.39
e	10	0.4	1.14	3.9 ± 0.73

^∗^Cumulative $\Delta$AEW over 150 min of processing. *DF*, dilution factor

### Effect of the process mode on LDH refolding

The online fluorescence monitoring was used to compare LDH refolding in pulsed mode to a single pulse dilution approach. Figure 2 presents the progression of intensity and AEW while Table 5 shows the resulting metrics in comparison to the KPIs of all processes.

The results presented in Fig. 2 show that information on the addition of protein pulses can be derived from the online intrinsic fluorescence signal. The intensity as well as the AEW steeply increased immediately after addition of solubilized protein indicating an increase of total protein concentration but in particular the reactive species of folding intermediates. The final shift in AEW of two experiments with the same dilution factor but different pulsing strategies (process b and process c) showed, that pulsed addition led to a higher final shift. This difference of 0.4 nm for the batch process also corresponds to a difference in specific activity that is 46% lower in the case of batch refolding (Table 5). The highest yields were achieved for the processes with high final dilution factors and late pulsing. These conditions avoided accumulation of the folding intermediates as low concentrations are added while the majority of the reaction within the previous interval has already finished [35]. Overall the results show, that pulsed addition of solubilized protein was better in terms of refolding yield and aggregation reduction than the batch refolding approach with a single addition of protein.

Figure 2C shows a higher *k* at lower protein concentrations. For pulsed refolding, the rate decreased for every pulse in correspondence to lower shifts of the AEW. These findings indicate a lower refolding rate at higher protein and GuHCl concentrations [35]. For pulsed addition, reactivity is the highest after the initiation of the processes. It is known that a critical parameter in a refolding process is the concentration of the denaturing agent and its ratio to the protein [20, 35]. As previously mentioned, the denaturant concentration must be high enough to reduce aggregation while at the same time still allow folding. Consequently, we assume that with further progression of pulsing (process a) a state of inertia would be reached where neither refolding nor aggregation would take place. In addition, both processes with a final dilution factor of 10, and the highest GuHCl concentration show low specific activities and a similar shift independent of the pulsing strategy (Table 5).

The delayed addition of solubilized protein to the refolding buffer in a so-called pulsed batch approach is a promising strategy to overcome low yields. Another benefit of this processing strategy is the possibility of increasing protein concentrations while simultaneously reducing aggregation [19, 35]. However, strategies for the intervals of addition are rather empirical. Thus, as depicted in Fig. 2, monitoring of intrinsic fluorescence is beneficial for proposing a pulsing strategy based on the observed end of refolding interval or a change in the folding rates across consecutive intervals.

### Online monitoring of GalOx and HRP refolding

**Fig. 3:**
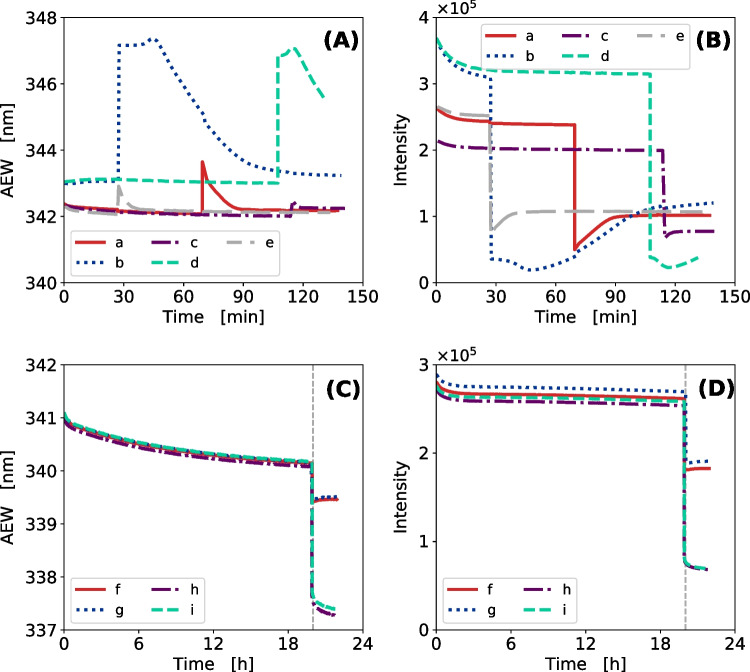
Online monitoring of GalOx and HRP refolding via intrinsic Trp and Tyr fluorescence. For GalOx refolding five different process modes (a–e) with variations in dilution factor and time of Cu(II) cofactor addition were monitored as the development of AEW (**A**) and fluorescence intensity integral (**B**) over time. Experiments were conducted in a DoE approach with the center point (process a) as biological replicates ($n=3$). Refolding of HRP was monitored via the change in AEW (**C**) and fluorescence intensity integral (**D**) during the process. HRP refolding at a protein concentration of 0.5 g L^-1^ was monitored over 20 h; subsequently, hemin cofactor was added to reach a hemin concentration of 5 (processes f and g) or 20 $\mu$M (processes h and i) and refolding was monitored for additional 2 h

To demonstrate the transferability of fluorescence-based monitoring of protein refolding, the refolding of two cofactor-containing enzymes, galactose oxidase (GalOx) and horse-radish peroxidase (HRP), was monitored (Fig. 3). The refolding runs of GalOx varied in the protein concentration and time of cofactor addition as shown in Table 6. The refolding runs of HRP varied only in the concentration of hemin cofactor added during the refolding (Table 6).

**Table 6:** Experimental design for GalOx and HRP refolding

GalOx refolding			
Process	DF	t_add_	Cofactor conc.
–	–	[h]	[mM]
a	30	1.25	1
b	10	0.5	1
c	50	2	1
d	10	2	1
e	50	0.5	1
HRP refolding			
Process	DF	t_add_	Cofactor conc.
–	–	[h]	[mM]
f	40	20	0.005
g	40	20	0.005
h	40	20	0.02
i	40	20	0.02

*t*_add_, time of cofactor addition; *DF*, dilution factor

For GalOx batch refolding processes with cofactor addition, similar trends were observed as for LDH refolding (“Effect of additives on LDH refolding”). Figure 3A shows that the progression of the refolding process correlated with the decreasing AEW, while the fluorescence intensity, in particular $F_0$, was in correlation with the amount of the total protein in solution (Fig. 3B). The shift in AEW varied for different dilution factors, depending on concentrations of protein and GuHCl (Fig. 3A). The refolding processes with the lowest dilution factor (process b and process d) resulted in the lowest refolding yield and did not show any changes in AEW prior to cofactor addition. This is in accordance with the absence of enzymatic activity measured at this stage of the process (Table S1). For GalOx, the addition of cofactor resulted in a steep increase in AEW of 5 nm and 1 nm (Fig. 3A), for the dilution factors 50 and 10, respectively. At the same time, the fluorescence intensity sharply dropped by 10-fold (Fig. 3B) which can be attributed to the quenching of fluorescence by copper ions [47]. Nevertheless, following copper addition, the online signal of AEW can be used to monitor conformational changes that are likely to be related to the formation of the thioether crosslink needed for full bioactivity [36]. Interestingly, the exponential decay of AEW after addition of the cofactor was up to 25-fold higher than prior to its addition. This indicated a rapid reaction that could be associated with the aerobic reaction mechanism of thioether bond formation [36]. Process b and process d, having the highest protein concentrations, showed a stagnation of AEW at 347 nm after cofactor addition (Fig. 3A). This phase was then followed by a decrease of AEW to the same range as prior to the cofactor addition with measurable exponential decay coefficients. A similar lag phase was observed in the changes of fluorescence intensity over time (Fig. 3B). In contrast to processes with lower dilution factor, the intensity of process b and process d stayed at a constant or even slightly declining level before the slow increase. We hypothesize that this behavior was caused by limitations in the cofactor concentration or inertia caused by the high GuHCl concentration. In both cases (processes b and d) low enzymatic activity was measured at the end of the refolding (Table S1).

The experimental design of HRP refolding was taken and adapted from Humer et al. [18]. Refolding of HRP at the protein concentration of 0.5 g L^-1^ was followed over 20 h before the hemin cofactor was added. The hemin cofactor was added to reach the concentration of 5 $\mu$M (Fig. 3C and D, processes f and g) or 20 $\mu$M (Fig. 3C and D, process h, i) and refolding was monitored for additional 2 h. The hemin cofactor addition was in all four runs accompanied with a sharp drop in AEW and intensity. This observation is likely a result of fluorescence quenching by hemin which causes the sharp drop in intensity but may also contribute to the shift in AEW. The conformational change of HRP upon the hemin addition was also probed using CD — Fig. S3A and B. The CD spectra suggest a small decrease in the $\alpha$-helical content of HRP upon the hemin addition, i.e., a slight decrease in CD signal at 222 nm. The sharp and intense drop in AEW is, therefore, likely to result from a combination of the HRP conformational change and the quenching of fluorescence by hemin which may be more pronounced for Trp fluorescence emission than for Tyr causing a blue-shift of AEW closer to fluorescence emission maximum of Tyr. The residual AEW and intensity drop over 2 h, apparent mostly in processes h and i, reflects the process completion. The drop in AEW and fluorescence intensity is much smaller for processes f and g in which the hemin concentration is only 5 $\mu$M compared to processes h and i with 20 $\mu$M hemin concentration. The difference in AEW and intensity after the hemin addition between processes f, g and h, i is likely to be caused by incomplete refolding due to insufficient cofactor concentration. This is in accordance with the observed HRP activity after the refolding process which is about one-third lower for processes f and g compared to processes h and i (Table S2).

**Fig. 4:**
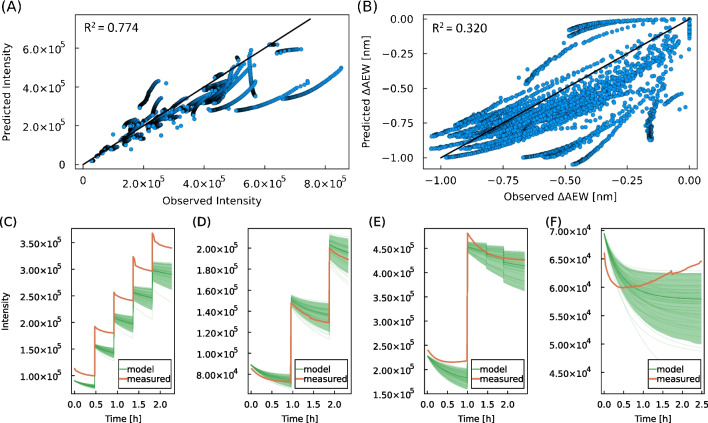
**A**–**B** Predicted vs observed for open-loop simulations of LDH refolding. The mechanistic model defined in “Process model” was solved with measured initial conditions after process start. The predicted *vs* observed plot is shown for (A) the fluorescence intensity with an $R^2$ value of 0.774 and (B) the $\Delta$AEW with an $R^2$ value of 0.320 ($n=38$ experiments). **C**–**F** Comparison of the fluorescence intensity integral between model and measurements for four distinct experiments (**C**)–(**F**). The ribbon of the model trajectory depict the standard deviation $\sigma$ of the estimation

It was shown that the online intrinsic Trp and Tyr fluorescence monitoring and the thereof derived quantifiable metrics (Fig. S1) can be applied to protein refolding processes with more complex protein structures, e.g., incorporating disulfide bonds or cofactor. The presented work shows the validity for observed correlations across three proteins differing in structural complexity and the number of disulfide bridges. For GalOx and HRP, conformational changes induced by the cofactor addition were observed. Thus, for a complex mechanism, as it is the case in protein refolding, the presented methodology poses a powerful PAT tool which enables better process understanding and therefore can be applied in industrial process development. This methodology is particularly promising due to its potential for scale-up and feasibility of monitoring the processes in larger industrial reactors using fiber optics and immersion probes. The experimental implementation of a fluorescence immersion probe to track HRP refolding is shown in Fig. S4 and described in SI “Online monitoring of GalOx and HRP refolding”.

### Usability of fluorescence monitoring in the model design

We set up a nonlinear mechanistic model for the protein refolding of LDH based on a first-order ODE system. Thereby, the different forms of protein conformation are usually simplified to a few model state variables, that are important for the evaluation of the general process dynamics. In this case the model considers three main folding states, the intermediate folding state (*I*), a native folding state (*N*) and aggregated protein (*A*). Even though this simplification comes with a certain loss of accuracy, it is still useful as it offers the possibility to describe the important dynamics of the process with few meaningful parameters. The mechanistic model was analyzed and adapted based on the obtained data. The two properties fluorescence intensity (*F*) and the change in average emission wavelength ($\Delta AEW$) were obtained from the intrinsic fluorescence spectra as measurements describing the process dynamics. As discussed above, the intensity shows a strong correlation to the protein concentration (Fig. S1) where the slight decrease of intensity over time at constant protein concentration is hypothesized to be due to the protein aggregation (“Effect of additives on LDH refolding”). Another factor contributing to the fluorescence intensity decrease over the timescale of refolding may be the protein adsorption to the surface of the cuvettes, however, this effect was not considered in the model. In terms of $\Delta AEW$ a correlation to the specific amount of intermediates (*I*) was assumed. In absence of a reliable measurement of intermediates, the best correlation to $\Delta AEW$ was found to be with the sum of *N* and *A* (Eq. 15), which is inversely correlated to *I*. Using these relationships in the mechanistic model, open-loop simulations were conducted for each refolding experiment. The model outputs *F*(*t*) and $\Delta AEW(t)$ were compared with the measured data from the intrinsic fluorescence in order to assess the quality of the model. The results were summarized in the form of a predicted vs observed plot shown in Fig. 4A–B.

As Fig. 4A–B show, the open-loop model exhibits $R^2$ values of 0.774 for the intensity and 0.320 for the $\Delta$AEW measurements. The inaccuracies of the model can be mainly explained by uncertain parameter values for the refolding kinetics and major measurement uncertainties in the offline protein state quantification [43, 48]. Furthermore, the model might not cover all the relevant process dynamics that are needed in order to represent the measured values more accurately.

These effects can be also observed in Fig. 4C–F where the prediction of four distinct processes are shown for the intensity. Whereas the prediction is shown to be accurate for some of the processes (D and E), there are also systematic offsets (C) as well as partly missing dynamics (F) occurring. Probably the simplified mechanistic model equations do not cover all effects that might occur during refolding as well as their impact on the fluorescence.

Still, the results presented in Fig. 4 show the potential of using the here developed grey box model for the prediction of protein states. Furthermore, it can be used to compute the most probable state estimate of the system using a state observer-like variations of the Kalman filter [12]. The presented modeling approach enables soft-sensing capabilities by estimating kinetic model parameters from online Tyr and Trp fluorescence measurements in real time.

## Conclusion

In this study, we implemented continuous *in situ* monitoring of protein refolding based on intrinsic tryptophan and tyrosine fluorescence for the first time. Using this technique, we were able to directly monitor the conformational change of the protein in the refolding process via the changes in the fluorescence maximum and intensity. We showed that neither the presence of chaotropic agents nor low protein concentrations limited the applicability of the method. The change of the fluorescence maximum during the refolding was expressed as AEW profile reflecting the changes in the center of mass of the fluorescence spectrum; the change in the signal intensity was expressed as the intensity integral over the emission spectrum measured. Quantifiable parameters were derived from the fitting of an exponential curve to the AEW profile and used for comparison between various refolding experiments in terms of the refolding kinetics, refolding efficiency and competitiveness between refolding and aggregation. The wide applicability and transferability of the method were demonstrated on the monitoring of the refolding of LDH with different additives and in different processing modes as well as on the refolding of two cofactor-containing enzymes, namely GalOx and HRP. The profile of AEW during the refolding was correlated to the amount of folding intermediates whereas the intensity integral was assumed to be partially related to the extent of aggregation. Both experimentally derived correlations were used as an input for a mechanistic protein refolding model, emphasizing the potential for state estimation methods based on intrinsic fluorescence in a real-time setting. A combination of online intrinsic fluorescence measurements and small-scale experiments gives insight into the refolding kinetics while maintaining the benefits of increased throughput. Moreover, the setup of the method is easily transferable to larger scale refolding, e.g., industrial refolding reactors, when employing fiber optics via the usage of immersion probes.

### Supplementary Information

Below is the link to the electronic supplementary material.

Supplementary file 1 (pdf 1036 KB)
