# Supplementary material for: Online monitoring of protein refolding in inclusion body processing using intrinsic fluorescence
Source: Anal Bioanal Chem. 2024 Apr 4;416(12):3019–32. doi: 10.1007/s00216-024-05249-1 (PMC11045631; doi:10.1007/s00216-024-05249-1)
Supplement: Supplementary file 1 — (pdf 1036 KB) [file 216_2024_5249_MOESM1_ESM.pdf]

Supporting Information

Online monitoring of protein refolding in inclusion body processing using intrinsic fluorescence

Chika Linda Igwe[1,2†], Don Fabian Müller[2†], Florian Gisperg[2,3], Jan Niklas Pauk[1,2], Matthias Kierein[2], Mohamed Elshazly[2,3], Robert Klausser[2,3], Julian Kopp[2,3], Oliver Spadiut[2,3], Eva Přáda Brichtová[2,3*]

[1]Competence Center CHASE GmbH, Hafenstraße 47-51, Linz, 4020, Austria.
[2]Research Area Biochemical Engineering, Institute of Chemical, Environmental and Bioscience Engineering, Technische Universität Wien, Gumpendorferstraße 1A, Vienna, 1060, Austria.
[3]Christian Doppler Laboratory for Inclusion Body Processing 4.0, Institute of Chemical, Environmental and Bioscience Engineering, Technische Universität Wien, Gumpendorferstraße 1A, Vienna, 1060, Austria.

*Corresponding author(s). E-mail(s): eva.prada@tuwien.ac.at;
Contributing authors: chika.igwe@tuwien.at;
fabian.mueller@tuwien.ac.at; florian.gisperg@tuwien.ac.at;
jan.pauk@tuwien.ac.at; e11778901@student.tuwien.ac.at;
mohamed.elshazly@tuwien.ac.at; robert.klausser@tuwien.ac.at;
julian.kopp@tuwien.ac.at; oliver.spadiut@tuwien.ac.at;
†These authors contributed equally to this work.

1 Quantifiable metrics for intrinsic Trp and Tyr fluorescence measurements

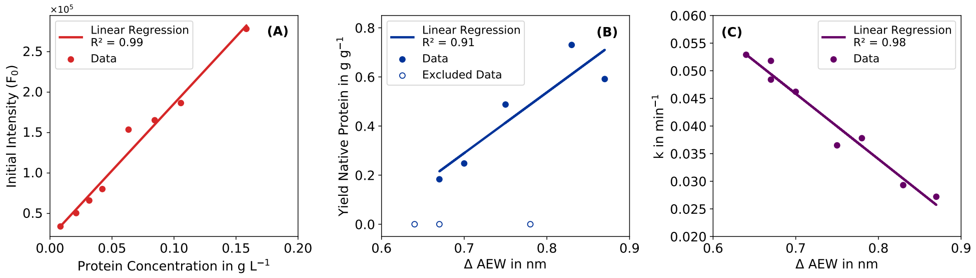

Fig. S 1 Comparison of KPIs between batch refolding processes at low concentrations. Protein refolding reactions were conducted at low concentrations between 0.01–0.18 g L^{-1}. A linear regression was used to correlate the data sets. (A) Intensity at t_0 over total protein concentrations in g L^{-1}. (B) Refolding yield in g g^{-1} over final ΔAEW in nm. Excluded data points refer to data sets, where concentrations fell below the sensitivity limit of the reference quantification method, thereby leading to 0 g g^{-1}. (C) Exponential decay parameter k in $\min^{-1}$ over final ΔAEW in nm.

The requirement for high sensitivity is a major challenge for the monitoring of refolding processes. Fig. S1 shows that measurements of the intrinsic fluorescence can lead to reproducible results at concentrations as low as 0.01 g L^{-1}, which is far below common limits-of-quantification of established PAT tools [1]. It can be assumed that the initial measurement of the intensity (F_0) corresponds to a condition where all proteins in solution are in an intermediate folding conformation [2]. Thus, assuming that all protein are still in solution and refolding and aggregation have not progressed yet, F_0 showed a strong correlation to the total protein concentration across separate LDH refolding processes (Fig. S1A). In addition, also the final shift in AEW correlated to the refolding yield and to the overall reactivity of the process depicted by the exponential decay coefficient of each curve (Fig. S1B and C).

To make processes comparable, usually quantifiable metrics, so called key performance indicators (KPIs), are used. For intrinsic fluorescence measurements in LDH refolding processes it was shown that the shift in AEW is an indicator for the state of the reaction. It can be assumed that it is caused by a decline of the reactive species, which are in this case the folding intermediates after initiation of the process. Consequently, as displayed in Fig. S1, the intensity in particular F_0 can be used as indicators for process parameters like protein concentration. In addition, the exponential decay parameter k characterizes the performance of LDH refolding as it depicts the progression of AEW shift over processing time and thus can be used to quantify the overall reactivity of the process. Those KPIs can be important for gathering process knowledge and for applications like process development or real-time monitoring of protein states. In addition they can be beneficial for model building with the final goal of real-time monitoring.

2 Effect of additives on LDH refolding

The initial intensity F_0 in Fig. S2A correlates with the various protein concentrations used in the refolding. However, higher drops in intensity occur at higher protein concentration, while the intensity in process d and process f maintained unchanged. Fig. S2B shows the typical exponential decay in AEW for all variations of buffer composition as observed for the other experiments. However, in the absence of refolding and aggregation, as it was the case for process a and process d, the AEW represents a straight line.

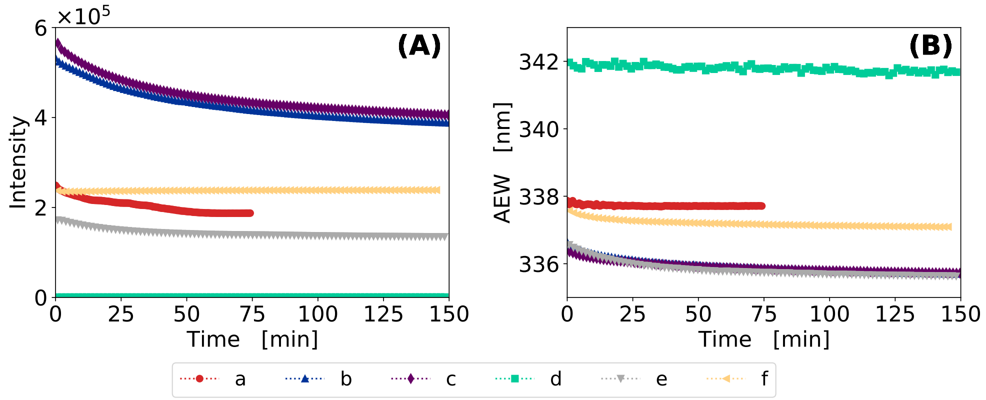

Fig. S 2 Intrinsic Trp and Tyr fluorescence measurements of LDH batch refolding processes with different additives. Six different process modes (a-f) with variations in buffer additive were monitored regarding (A) integral of intensity and (B) shift in average emission wavelength in nm.

3 Online monitoring of GalOx and HRP refolding

3.1 Quantifiable metrics GalOx refolding

Tab. S 1 Comparison of KPIs of GalOx refolding

Process	DF	t_{add}	Specific Activity	Aggregation	*k1*	*k2*	$\Delta AEW1$	$\Delta AEW2$
	-	[min]	[U mg^{-1}]	[g g^{-1}]	[min^{-1}]	[min^{-1}]	[nm]	[nm]
a†	30	75	0.90 ± 0.19	0.16 ± 0.03	0.0242	0.1248	0.36	1.44
b	10	30	0.40 ± 0.02	0.28	ND	0.034	ND	1.84
c	50	120	1.31 ± 0.01	0.117	0.0272	0.6792	0.42	0.23
d	10	120	0.33 ± 0.11	0.32	ND	0.0185	ND	0.11
e	50	30	1.19 ± 0.02	0.09	0.1225	0.2344	0.21	1.03

†mean of biological replicates, ND: not detectable, DF: dilution factor, *k1* and $\Delta AEW1$: prior to Cu^{2+} addition, *k2* and $\Delta AEW2$: after Cu^{2+} addition

3.2 HRP activity assay

Tab. S 2 HRP activity assay

Process	HRP concentration	Hemin concentration	Volumetric activity*	$\Delta AEW1$	$\Delta AEW2$
	[g L^{-1}]	[μM]	[U mL^{-1}]	[nm]	[nm]
f	0.5	5	41.4 ± 6.2	0.92	0.68
g	0.5	5	39.9 ± 12.1	0.91	0.65
h	0.5	20	66.5 ± 12.5	0.94	2.81
i	0.5	20	61.5 ± 8.0	0.93	2.78

*The volumetric activity was tested for the samples at the end of monitored refolding process, i.e. after 22 hours of refolding. The average volumetric activity and its standard deviation for each process was calculated from 9 independent activity measurements.
$\Delta AEW1$: prior to hemin addition, $\Delta AEW2$: after hemin addition.

3.3 HRP refolding – hemin cofactor addition

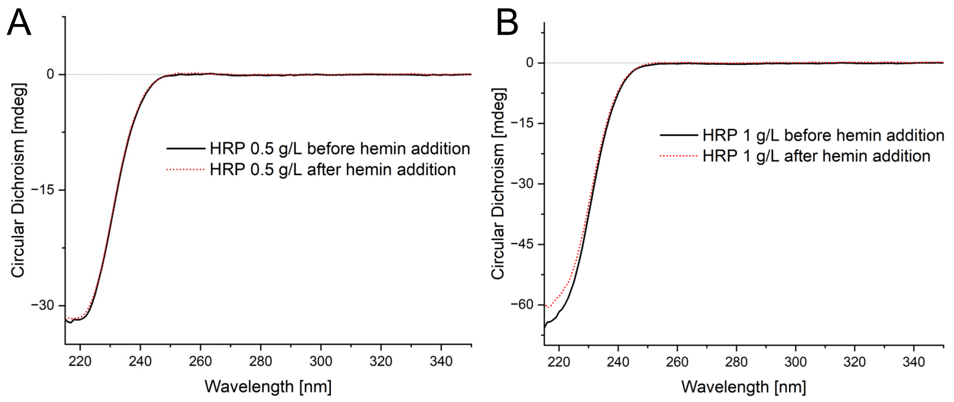

Fig. S 3 Circular dichroism spectra of HRP after 20 hours of refolding before and after hemin addition. HRP was refolded for 20 hours at 0.5 g/L (A) and 1.0 g/L (B) concentration in a buffer containing 2 M urea, 2 mM $CaCl_2$, 7 % (v/v) glycerol, 1.27 mM GSSG, pH 10. After the initial refolding period hemin cofactor was added to reach 20 μM hemin concentration in the sample. Circular dichroism spectra were recorded directly before and immediately after the hemin addition.The temperature of a cuvette holder during the spectra measurement was set to 5°C.

3.4 HRP refolding – online monitoring with immersion probe

The first feasibility experiment of online fluorescence monitoring of refolding in larger volumes/containers was performed using an immersion probe. The measurement was conducted using an FP-8550 Spectrofluorometer (Jasco, Tokyo, Japan) equipped with an optical fibre unit OBF-132 (Jasco, Tokyo, Japan) and a custom-made fluorescence fiber optics probe FlexiSpec® (art photonics GmbH, Berlin, Germany). Refolding was carried out in the volume of 4 mL in a glass vial with a magnetic stirrer (Fig. S4B). The vial was immersed in an ice-bath to ensure the cooling (Fig. S4C). The sample was covered with an aluminium foil to ensure the light protection during the measurement (Fig. S4D). The sample was excited at 280 nm and the emission spectrum was recorded between 310–370 nm with a step size of 0.5 nm. Excitation and emission slits of 2.5 nm and 10 nm, respectively, were used. The scanning speed was set to 200 nm min^{-1}, sensitivity to high, and the response time to 1 s. Data processing was conducted analogously to the cuvette-refolding. The monitoring was tested on the first 1.2 hours of HRP refolding (Fig. S4A).

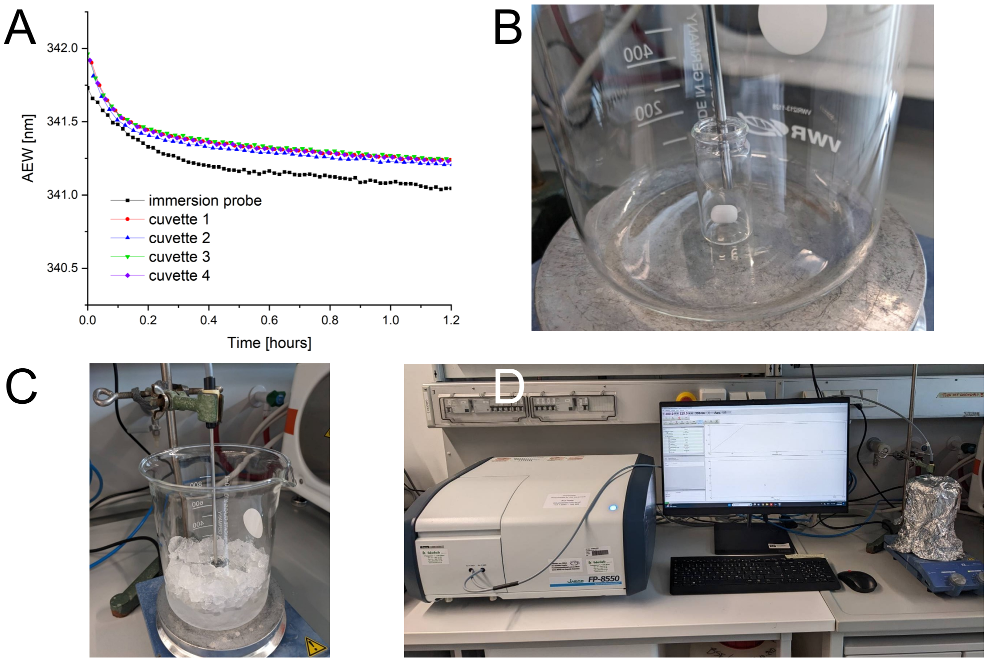

Fig. S 4 Online monitoring of HRP refolding using immersion probe set-up. The first 1.2 h of the initial phase of HRP refolding were monitored using an immersion probe set-up. AEW profile recorded using the immersion probe (in black) was overlaid with the first 1.2 hours of HRP cuvette-refolding profiles shown in Fig. 3C (A). The HRP refolding monitored by immersion probe was performed in a glass vial with a magnetic stirrer in the volume of 4 mL (B). The vial with the refolding sample was immersed into the ice-bath to ensure the cooling during the refolding (C). In the final instrumental set-up (in D), the ice-bath was covered with an aluminium foil to prevent the light interference during the measurement. The identical refolding buffer composition as for the cuvette-refolding was used.

References

[1] Pauk, J.N., Raju Palanisamy, J., Kager, J., Koczka, K., Berghammer, G., Herwig, C., Veiter, L.: Advances in monitoring and control of refolding kinetics combining PAT and modeling. Applied Microbiology and Biotechnology **105**(6), 2243–2260 (2021) https://doi.org/10.1007/s00253-021-11151-y

[2] Buscajoni, L., Martinetz, M.C., Berkemeyer, M., Brocard, C.: Refolding in the modern biopharmaceutical industry. Biotechnology Advances **61**, 108050 (2022) https://doi.org/10.1016/j.biotechadv.2022.108050
